# O3-4 Progress on addressing the cornerstones of physical activity policy for children and youth in England

**DOI:** 10.1093/eurpub/ckac094.020

**Published:** 2022-08-29

**Authors:** Karen Milton, Anna Chalkley

**Affiliations:** 1 Norwich Medical School, University of East Anglia, Norwich, United Kingdom; 2 Loughborough University, Loughborough, United Kingdom

**Keywords:** Physical activity, Policy, Recommendations, Surveillance, Public education

## Abstract

**Background:**

There has been an increasing focus on the importance of national policy to address population levels of physical inactivity. It has been suggested that the four ?cornerstones' of policy comprise: 1) national guidelines on physical activity levels; 2) setting population goals and targets; 3) surveillance or health monitoring systems; and 4) public education. The aim of the current paper was to analyse the policy actions which have addressed each of these elements for children and youth in England and to identify areas of progress and remaining challenges.

**Methods:**

A literature search was undertaken to identify past and present documents relevant to physical activity policy for children and youth in England. Each document was analysed to identify content relevant to the four ?cornerstones' of policy.

**Results:**

Physical activity guidelines for children and youth have been in place since 1998 and reviewed periodically to ensure they reflect the latest scientific evidence. The setting of physical activity targets has focused on the provision of opportunities for physical activity, particularly through physical education (PE) in schools, rather than in relation to the proportion of children meeting recommended physical activity levels. There has been much surveillance of children's physical activity but this has been undertaken infrequently over time, by a wide range of organisations, and with varying inclusion of different domains of activity such as school PE, leisure time activity and active travel. There has only been one campaign in England targeted at children and their intermediaries (Change4Life), which was an obesity campaign focussing on dietary behaviour in combination with physical activity. Most recently an infographic supporting the physical activity guidelines for children and young people was developed, but details of its dissemination and usage are unknown.

**Conclusions:**

There have been many developments in physical activity policy in England targeted at children and youth. The area of greatest progress is national physical activity guidelines. Establishing prevalence targets, streamlining surveillance systems, and investing in public education would strengthen national policy efforts to reduce physical inactivity.

